# Development and validation of a machine learning model for early prediction of intensive care unit acquired weakness

**DOI:** 10.1186/s40635-025-00810-3

**Published:** 2025-09-30

**Authors:** Felipe Kenji Nakano, Nathalie Van Aerde, Grégoire Coppens, Ilse Vanhorebeek, Celine Vens, Greet Van den Berghe, Fabian Güiza Grandas

**Affiliations:** 1 Department of Public Health and Primary Care, KU Leuven KULAK, Etienne Sabbelaan 53, 8500 Kortrijk, Belgium; 2 Itec, imec Research Group at KU Leuven, Etienne Sabbelaan 51, 8500 Kortrijk, Belgium; 3 Department of Cellular and Molecular Medicine, Clinical Division and Laboratory of Intensive Care Medicine, KU Leuven, UZ, Herestraat 49, 3000 Louvain, Belgium

**Keywords:** Supervised Machine Learning, Intensive Care Unit, Random forest, Critical illness, Muscle weakness

## Abstract

**Background:**

Early identification of potential high cost and high need patients on the ICU may assist in the development of targeted protocols, which allows proper resource utilization and initialization of preventive care. Weakness acquired in the ICU developed within the first week is an independent predictor of both short and long-term adverse outcomes, nonetheless early prediction is challenging. We aimed to develop and validate a machine learning model for ICU acquired-weakness (ICU-AW), using data readily available within the first 24 h of ICU admission.

**Methods:**

Patients from the EPaNIC trial (NCT00512122, *N* = 4640) who were assessed for muscle weakness at day 9 (IQR 8–13), after ICU-admission, using the Medical Research Council (MRC) sum. Patients are diagnosed with ICU-AW if their MRC is lower than 48. The final subset contains *N* = 600. Our models were internally validated using 100 repetitions of fivefold cross validation. We compared three predictive models: (i) a random forest and (ii) a logistic regression model built using descriptors available at day 1, (iii) a random forest using only APACHE II as a descriptor. Both random forests contain 150 trees.

**Results:**

The training set comprised 600 patients where the incidence of ICU-AW was 38.6% (232/600). The AUROC of the random forest with all descriptors and the logistic regression were 76% and 74%, respectively. The random forest (RF) achieved a specificity of 62% and a sensitivity 79%, whereas the logistic regression yielded 69% and 68%, respectively. The RF identified APACHE II, creatinine, SOFA PaO2/FiO2, bilirubin, BMI, age, glycemia upon admission, morning glycemia and sepsis as the most relevant descriptors. Lastly, the RF also presented very good calibration and clinical usefulness for a wide range of risk thresholds.

**Conclusions:**

Machine learning models, especially random forests, can be used to predict if patients are at risk of developing ICU-AW, using data available within 24 h of admission. This tool allows prognostication early in an adult general critically ill patient population, with the potential to detect high cost and high need patients who benefit from different levels of care.

**Supplementary Information:**

The online version contains supplementary material available at 10.1186/s40635-025-00810-3.

## Introduction

Health care providers are under increasing pressure to deliver care that is aligned with both socio-economical demands and patient goals [1–5]. Typically, outlier patients require more resource utilization [6]. Ensuring high value care in a sustainable format depends on proficient identification of these “high cost and high need” patients and delineate from which interventions they benefit [3, 5]. In an intensive care unit (ICU), patients who develop intensive care unit acquired weakness (ICU-AW) represent a patient phenotype with particularly inflated downstream costs in both the short and long-term [7–9].

ICU-AW is characterized by a generalized and symmetrical deterioration of respiratory muscle and limbs, which is associated with a significant loss of muscle tone and mass [10]. It also leads to prolonged mechanical ventilatory support [11], higher chances of extubation failure [12], higher mortality risk up to 5 years [13], reduced walk and exercise ability and overall worse quality of life [13]. Currently, no effective treatments are available for ICU-AW [14].

The most used method to diagnose ICU-AW, the medical research council (MRC) scale, consists of manually assessing the strength of different muscles groups [15]. This procedure requires patients to be responsive and cooperative and assessors to have established inter-observer agreement of scoring [14].

We hypothesize that machine learning models can, at day 1, reliably predict whether patients are at risk of developing ICU-AW by day 9. This will provide physicians with prediction regardless of whether the patient can be diagnosed via the MRC scale, enabling the initiation of preventive care and proper resource utilization. Furthermore, identifying patients at particular risk for ICU-AW would be interesting for possible new interventional studies, such as: use of electrical muscle stimulation; highly restrictive use of glucocorticoids and use of neuromuscular blockers.

## Methods

### Study design

The training and internal validation cohort initially consisted of the subgroup of patients recruited during the “Impact of Early Parenteral Nutrition Completing Enteral Nutrition in Adult Critically Ill Patients”, EPaNIC-trial (NCT00512122, *N* = 4640). The inclusion and exclusion criteria are available in the Supplementary Material, Section 2.

This dataset was further filtered to include only patients that underwent ICU-AW assessment on day 9 (IQR 8–13), from ICU-admission [16]. As reported in [16], patients were excluded if they were never awake, displayed medical instability, had pre-existing neuromuscular disease, could not communicate with the assessor due to language barrier, could not be assessed due to unavailability of assessors, or simply declined participation.

This study was performed in accordance with the protocol as described in the European Medicine Agency's "Note for guidance on good clinical practice CPMP/ICH/135/95." as well as the Declaration of Helsinki [17]. The resulting dataset contains 600 patients.

Clinical strength was prospectively assessed by physiotherapists with known good interrater reliability at general ICU- wards at the university hospitals of Leuven, Belgium, in populations admitted in from December 2008 to November 2010, excluding patients with known prior neuromuscular disorders.

As recommended in [18], we report the “Key Reporting Metrics” in the Supplementary Material, Table 2.

### Outcome of interest

The outcome of interest is ICU-AW measured using the medical research council sum scale [15]. More specifically, it consists of a bedside bilateral assessment of strength of 6 muscles groups, namely: shoulder abduction, elbow flexion, wrist extension, hip flexion, knee extension, foot dorsiflexion. That is, each category of muscle receives two scores, one per each limb, from 0 (no contraction) to 5 (full muscle force). The final diagnosis is determined by the summed score, if its value is lower than 48, then the patient has developed ICU-AW.

### Modelling strategy

We followed the TRIPOD guidelines for developing multivariable models [19]. Candidate predictor variables used as input for the model were preselected by experts in the field based on estimated importance as risk factors for weakness, and by availability in electronic health records upon admission [16, 20–23]. More specifically, we have included baseline factors (age, diabetes, BMI as a continuous value, malignancy, sepsis, ge`nder and preadmission dialysis), admission factors (infection upon admission, APACHE II, admission glycemia), factors reflecting the first 24 h of ICU-stay (laboratory values as markers for severity of individual organ failure, i.e., total serum bilirubin, CRP, serum creatinine, glycemia levels at admission and in the morning after, and SOFA PaO2/FiO2) and treatment factors (mechanical ventilation and corticosteroid administration on the first day). All features were used as input in their original form.

We used three predictive models in total: (i) a random forest and (ii) a logistic regression, both built using all descriptors available at day 1 and (iii) a random forest built using only APACHE II as a single descriptor. We added the third model to assess the potential non-linearity between APACHE and ICU-AW and to assess whether other descriptors in addition to APACHE would improve the predictive performance.

We have used threefold internal cross-validation to optimize the number of trees in the random forest, considering the following values {50, 100, 150, 200}. The value 150 was consistently chosen as the best parameter. The logistic regression was trained using L2 normalization. All other parameters were set to default. The features were standardized for the logistic regression.

Missing values were imputed using multiple chained equations with 31 iterations [24]. The most relevant features were selected according to the inherent feature importance measured using impurity reduction.

We have employed Python version 3.10.12, Numpy 1.24.1 and Pandas 1.5.3. Graphs were generated using Matplotlib 3.7.1, Pycaleva 0.7^1^ for calibration curves and Dcurves 1.0.6 for decision curves.^2^

### Validation

Prediction of ICU-AW was internally validated in the development cohort using 100 repetitions of fivefold cross validation. That is, patients were randomly split into development and testing subsets without intersections. We report the average results and the standard deviation considering the 500 folds.

### Evaluation criteria

The model was evaluated using the area under the receiver operating curve (AUROC), sensitivity and specificity. The threshold values used for sensitivity and specificity were obtained using the Youden index [25]. For all three measures, the higher the value, the better the discriminatory performance of the model, such that 100% corresponds to perfect discrimination.

To assess the reliability of our model, we have measured the calibration of our model using the calibration belt [26], which can be visualized as a 2-dimensional plot. If the diagonal of the plot is within the confidence interval (blue area), then the model is considered to be well-calibrated (*p*-value larger than 0.05).

Furthermore, we have also assessed clinical usefulness using decision curves [27]. A decision curve describes the clinical usefulness of our model by visualizing its (net) benefit above or below the standard medical practice. The net benefit corresponds to the relative harm and benefit that a certain decision has, which is measured using Eq. 1, where *A* stands for the number of patients correctly classified by the model as developing ICU-AW, *B* is the number of patients wrong classified by the model as developing ICU-AW, *pt* is the threshold being applied and *N* is the total number of patients.

1 $$\frac{\left(A - B \cdot \left(\frac{pt}{1-pt}\right)\right)}{N}$$

Net benefit used to measure the clinical usefulness.

### Online application

We have developed an online application, which is publicly available,^3^ with an adapted version of the random forest where only the 10 most relevant features, which were selected using feature importance, ("Most relevant descriptors" section) are employed. We have used the same resources listed in "Modelling strategy" section and also the libraries Shiny 0.6,^4^ Shap 0.43 [45] 5 and Scikit- learn 1.3.2.^6^

### Ethical consent

The EPaNIC study protocol and informed consent forms were approved by the Leuven University Hospital Ethics Committee (ML4190) in 2007.

## Results

### Dataset

As can be seen in Table 1, 600 patients had MRC scores measured: 232 (39%) patients were diagnosed with ICU-AW, 368 (61%) did not develop ICU-AW. Patients with ICU-AW, as compared to those without weakness, were older, more likely to have sepsis or infection upon admission, more likely to have received steroids, presented higher SOFA PaO2/FiO2 and APACHE II score and higher CRP and creatinine levels. Histograms built using the laboratory factors, admission glycemia, bilirubin, C-reactive protein, creatinine and morning glycemia, are available in the Supplementary Material, Sections 1.7, Figs. 8, 9, 10, 11 and 12.

**Table 1:** Descriptive analysis of the dataset

	#ICU-AW (*n* = 232)	# Non ICU-AW (*n* = 368)	*P*-value
Baseline Factors			
Age (0%, *N*)	63.36 (13.57)	60.23 (16.26)	0.014
Diabetes (0%, *B*)			1
Yes	37 (16%)	59 (16%)	
BMI (0%, *N*)	26.61 (6.55)	26.12 (4.85)	0.29
Malignancy (0%, *B*)			0.51
Yes	67 (29%)	96 (71%)	
Sepsis (0%, *B*)			< 0.001
Yes	141 (61%)	123 (33%)	
Gender (0%, *B*)			0.49
Male (1)	100 (43%)	147 (40%)	
Dyalisis pre-admission (0%, *B*)			0.32
Yes	4 (2%)	2 (1%)	
Admission Factors			
Infection upon admission (0%, *B*)			< 0.001
Yes	147 (63%)	129 (35%)	
APACHEII (0%, N)	34.1 (8.79)	25.85 (9.79)	< 0.001
Admission glycemia (1%, *N*)	145.26 (60.48)	144.42 (49.03)	0.14
24 h ICU stay Factors			
Bilirubin (3%, *N*)	1.88 (3.84)	1.50 (3.66)	0.19
C-Reactive protein (3%, *N*)	151.59 (125.39)	97.24 (92.31)	< 0.001
Creatinine (3%, *N*)	1.83 (1.40)	1.31 (1.42)	< 0.001
Morning glycemia (1%, *N*)	114.00 (45.2)	109.2 (36.12)	0.85
SOFA PaO2/FiO2 (1%, *N*)	190.15 (92.83)	239.79 (95.7)	< 0.001
Treatment Factors initiated at ICU admission			
Mechanical ventilation support (0%, *B*)			0.47
Yes	217 (94%)	337 (92%)	
Steroids (0%, *B*)			< 0.001
Yes	74 (68%)	65 (18%)	

Each descriptor is further described by their percentage of missing values. Furthermore, numerical descriptors (*N*) are described using their mean and standard deviation, whereas binary descriptors (*B*) are described using absolute values and their corresponding percentage

### Model performance

**Fig. 1:**
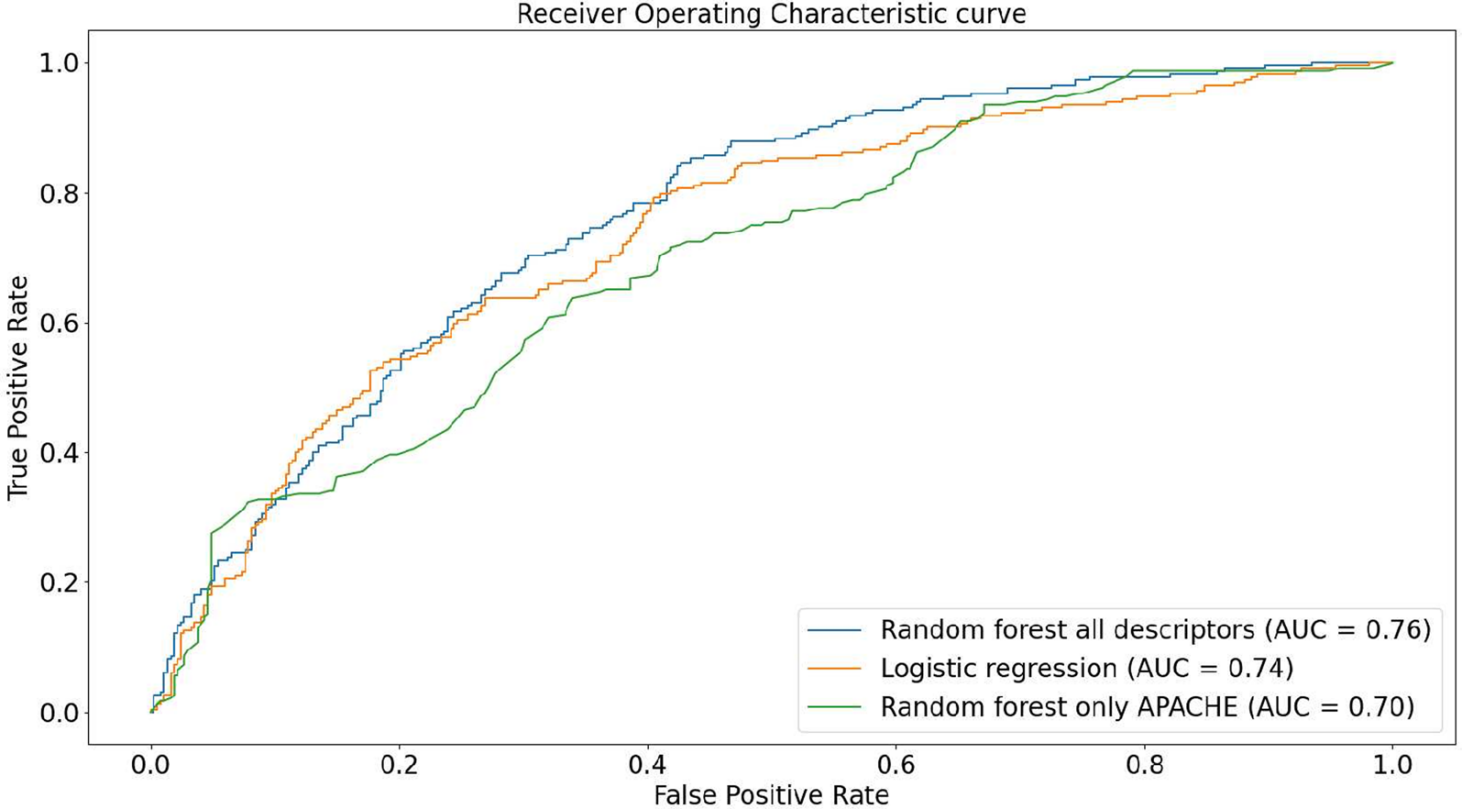
ROC curve considering fivefold cross-validation. The x-axis contains false positive rate, whereas the y-axis contains the true positive rate

As presented in Fig. 1, the AUROC of the random forest using all descriptors was 76%, whereas the logistic regression reached 74%. The random forests using only APACHE II yielded 70%. In terms of specificity and specificity, a similar behavior is observed where the random forest with all descriptors presents 62% and 79%, respectively, in comparison to the random forest using only APACHE II (63% and 67%). The logistic regression reached 69% in specificity and 68% in sensitivity. All models presented standard deviation smaller than 0.01, regarding all measures. The thresholds selected by the Youden index for the LR averaged 0.39 with standard deviation of 0.04, ranging from 0.27 to 0.49. Similarly, the thresholds selected for the RF averaged 0.37 with standard deviation of 0.03, ranging from 0.29 to 0.44. These results are summarized in Table 2.

**Table 2:** Average results obtained on 100 times fivefold cross validation

	RF	LR	RF APACHE II
AUROC	76	74	70
Specificity	62	69	63
Sensitivity	79	68	67

RF stands for the random forest with all descriptors, LR for logistic regression and RF APACHE II for the random forest using only APACHE

**Fig. 2:**
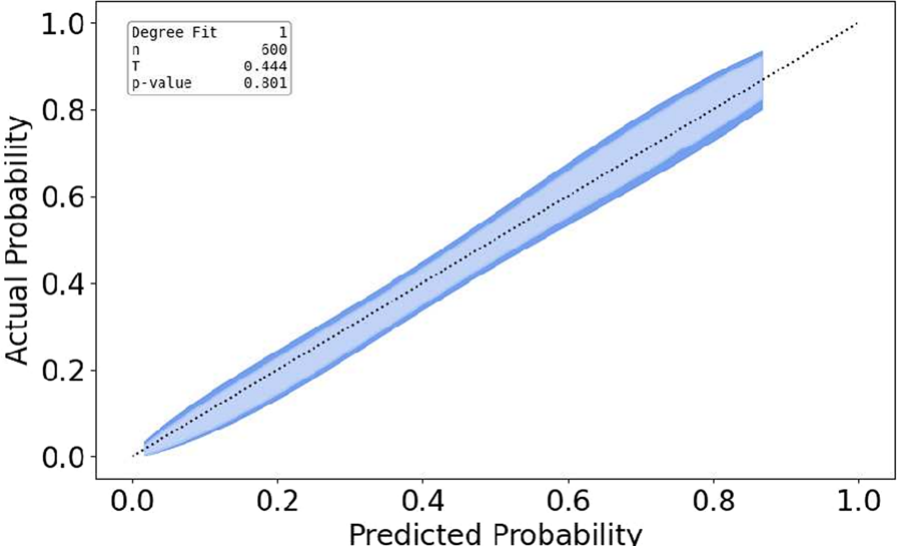
Calibration belt considering fivefold cross-validation for the random forest. The x-axis contains the probabilities predicted by the random forest, whereas the y-axis contains the expected output. In this case, no evidence of miscalibration can be found since the diagonal is within the blue region

**Fig. 3:**
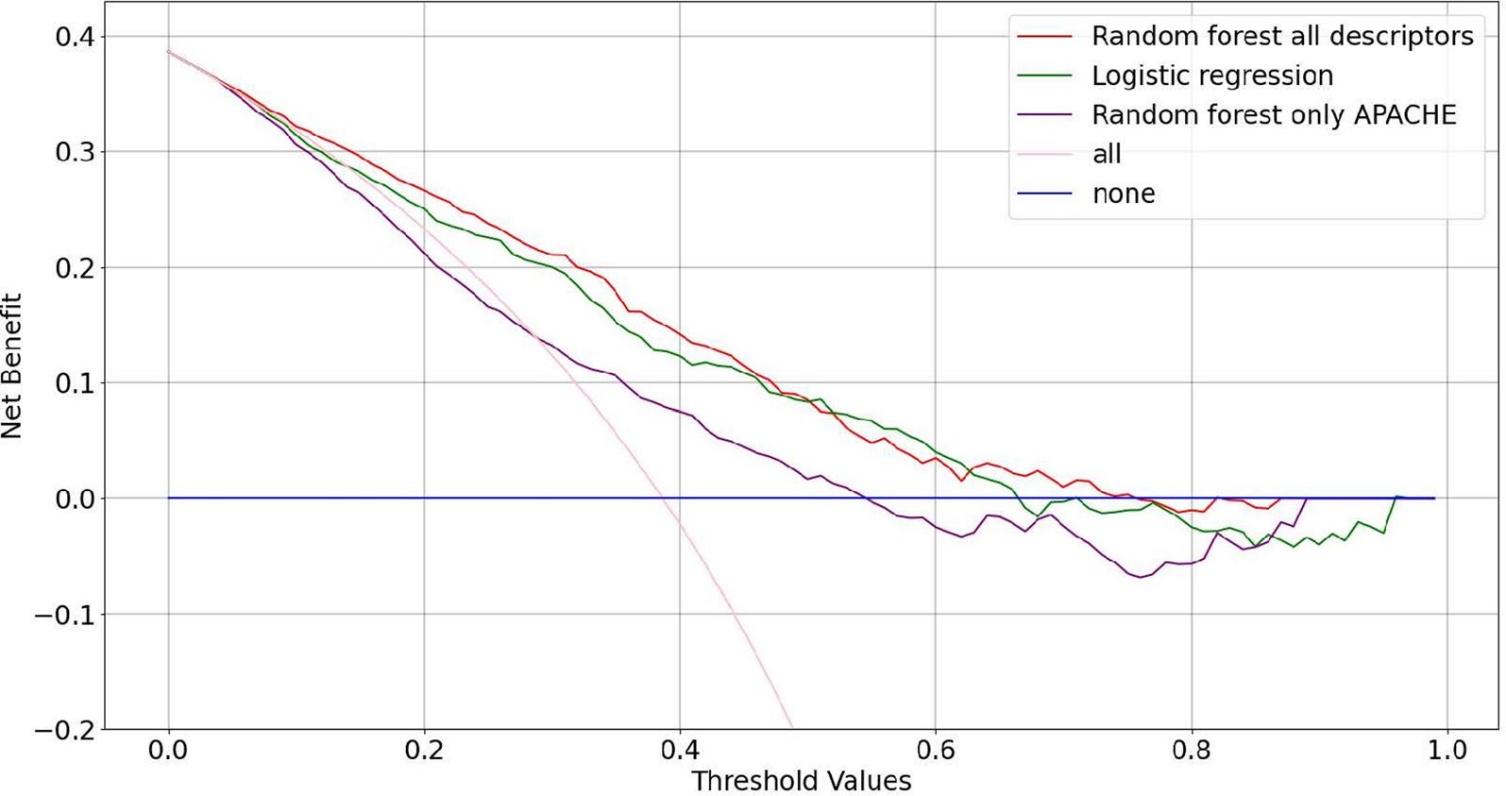
Decision curve considering fivefold cross-validation. The x-axis contains the probabilities predicted by the random forest, whereas the y-axis contains the clinical usefulness (net benefit, Eq. 1). In this case, clinical usefulness is observed for the random forest in thresholds ranging from 10 to 70%

Furthermore, the random forest with all descriptors presented very good calibration, as shown in the calibration belt in Fig. 2, which contains the diagonal for the entire predicted probability range. The decision curve (Fig. 3) indicated clinical usefulness with net benefit above the default strategies for thresholds ranging from approximately 10% to 70%, for the random forest with all descriptors. The calibration belt of the logistic regression, the random forest using only APACHE II are available in the Supplementary Material Figs. 6 and 7, respectively. Results obtained using a random forest and SOFA PaO2/FiO2 as a sole descriptor are available in Supplementary Material Table 1. Its calibration belt is available in the Supplementary Material Fig. 7.

### Most relevant descriptors

The random forest has identified the following 10 descriptors as the most relevant: APACHE II, Creatinine, SOFA PaO2/FiO2, C-Reactive protein, bilirubin, BMI, age, morning glycemia, glycemia at admission and sepsis upon admission. A complete ranking of descriptors is available in the Supplementary Material Sect. 1.1.

### Online application

Using a fivefold cross validation, our publicly available online application,^7^ with an adapted version of our model where only the 10 most relevant features ("Most relevant descriptors" section) are employed, reached 74% of AUROC, which is a minimal difference in comparison to using all predictor variables (76%). Similarly to the random forest using all descriptors, the random forest available in the web application is well calibrated, as seen in the calibration belt in Supplementary Material Figure 3. Further, regarding clinical usefulness (Supplementary Material Figure 4), it also presents net benefit above the default strategies when thresholds ranging from approximately 10% to 70% are used.

More information about the online application is available in the Supplementary Material Sect. 1.2.

## Discussion

In this study, we evaluated the use of machine learning models for early prediction of ICU-AW. To ensure long-term sustainability of health care systems, tailored treatment is increasingly enforced through an accountability burden for treatment choice. As costs tend to concentrate in selected patient groups, it may be necessary to devise specific protocols for these outliers [28]. In ICU, resource usage is the highest in long-stay patients, and among them, patients who will develop intensive care unit acquired weakness (ICU-AW) represent a subgroup with particularly inflated costs [29].

In addition, interventions prescribed for this patient group, such as long-term recovery of premorbid physical function and quality of life, are not necessarily successful [9, 29–33], ensuring high value care will increasingly depend on ability to identify the “high cost and high need” patients, and delineate from which interventions they benefit the most [2, 4, 28, 34].

ICU-AW is a well-known predictor of both short and long-term outcome in ICU- patients [9, 29, 35, 36]. Upfront patient stratification by risk of ICU-AW as surrogate marker for individual patient trajectory could guide proactive treatment planning. As such, it holds added value over ICU-AW assessment itself. Indeed, the current body of evidence linking ICU-AW to outcome focused on its presence or absence on day 9, which indicates the beginning of prolonged critical illness [13, 30]. The clinical testing of peripheral muscle strength is inexpensive and simple, but reproducibility depends on assessment by trained health care providers: systematic daily assessment of all ICU-patients by the same trained physiotherapists is not a feasible clinical reality. Conversely, delayed formal ICU-AW assessment until clinical suspicion due to its associated complications (e.g. delayed weaning) or due to lack of cooperation (e.g., due to sedation, use of neuromuscular blocking agents, delirium) precludes the possibility to anticipate the ICU-trajectory upfront, and impedes targeted patient inclusion in randomized controlled trials aiming to reduce the burden of ICU-AW.

We created a model for predicting ICU-AW using a cohort of adult critically ill patients demographically representative of a general ICU population and made it available online. We based our prediction on clinical ICU-AW assessments performed by the same trained physiotherapists in an internal validation setup. Using a random forest, we constructed an accurate and calibrated model using baseline characteristics and descriptors readily available within 24 h after ICU admission at the bedside.

Based on its inherent feature importance, the random forest identified APACHE as the most relevant descriptor. This was expected, as it combines several factors that are related to the development of weakness, such as: high severity of illness implying high likelihood of sepsis and high likelihood of prolonged mechanical ventilation, use of steroids and neuromuscular blocking agents, among others.

However, age and creatinine, which are already included in the APACHE, are surprisingly descriptive. We can interpret this as an indication that pre-ICU frailty (i.e., higher creatinine likely captures patients with preexisting renal disease) is a very strong predictor of more frailty and more adverse ICU outcomes. This could also be related to the non-linear relationship between creatinine and ICU-AW, when taking into account interactions with covariates such as APACHE II, as can be seen in the partial dependency plot presented in Supplementary Material Fig. 13.

This is in line with the perceived evolution in the task of critical care physicians, namely to include a long-term perspective in their care and incorporate clearly established predictors of ICU and post ICU disability in early goals of care discussions for patients likely to not do well both in the ICU and beyond [37].

Our research has several strengths. First, our modelling approach has several methodological advantages when compared to currently available prediction models for ICU-AW. Namely, we rely on well-established machine learning models, whereas the other studies use step-wise logistic regression, which is inherently flawed [38]. Moreover, other models use data collected up until ICU-AW evaluation, such as duration of mechanical ventilatory support, to increase accuracy for detection of prolonged critically ill patients [21, 22]. Alternatively, they depend on data available only at day 2 after admission [23]. Thus, they employ data collected throughout the disease course, which excludes or reduces the possibility of predicting the outcome upfront and consequently of early initiation of preventive care. Our model uses variables which are frequently recorded in most of the ICUs and are also extractable from electronic health records.

Second, our study reported not only AUROC, sensitivity and specificity, but also model calibration and decision curves (clinical usefulness), as opposed to other studies [39, 40].

Third, as shown in the decision curve (Fig. 3), our model shows benefits above default strategies in the risk range below 0.5. Hence, using a relatively low threshold would be advantageous, as it increases the number of correctly identified ICU-AW in the expense of few incorrectly diagnosed with ICU-AW. For instance, if we take a false negative cost three times as high as a false positive cost, our model yields 88% sensitivity and 53% specificity, compared to 84% and 53% for the logistic regression model, respectively. This is particularly interesting for the inclusion of patients in new RCTs aiming to prevent ICU-AW or to initiate goals of care discussions in patients expected to have very poor long-term outcome based on this model among others. This allows the implementation of more efficient RCTs to assess the effect of interventions, since most of them still have limited success [14].

Lastly, our experiments included a larger number of descriptors, which enables us to assess their influence on ICU-AW. In principle, this could be a hindrance to some machine learning models, nonetheless our employed model, random forest, can correctly handle and benefit from such higher number of descriptors, as it is robust to overfitting [41]. Further, we showcased that using all descriptors is superior to using solely APACHE II. Our model is also available as an online application, which allows clinicians to estimate model performance in a sample of their patients, compensating for the lack of formal external validation. Since external validation will likely require a large university center, our online application allows physicians working in smaller hospitals to assess the applicability of the model in their patient populations.

Our study has important limitations. The choice of an early prediction model, using data limited to the first 24 h of ICU-stay, inherently limits the expected optimal performance for prediction of ICU-AW and long-term outcome [30]. To ensure that our model was informative of the long-term trajectory without including temporal indicators reflecting the in-ICU trajectory, we validated our model to predict weakness at day 9 of ICU-stay, as this time frame was shown to represent the cut-off towards prolonged critical illness known to be related to incremental adverse long-term prospects.

Another potential limitation of our study is the use of the MRC sum score to diagnose ICUAW, while a two-tier approach using handgrip dynamometry and a through range 4-grade score showed better inter-operator agreement for ICUAW diagnosis [42]. Nevertheless the MRC sum score remains the most used diagnostic procedure for ICUAW [14] and has demonstrated good inter-operator agreement [43, 44], while the two-tier approach still requires further validation.

Thirdly, ICU-AW was only assessed on the patients who were awake and cooperative at day 9, which can lead to possible bias, as competing factors, such as mortality prior to day 9, were not taken into account. Furthermore, the clinical trial from which the data originates dates to 2010, while different patient management strategies such as different types of sedation and variations in feeding strategies might be currently used.

Lastly, our experiments were limited to a single-center. External validation is thus required to assess the validity of the model in other centers. However, the online application tool allows clinicians to estimate the out-of-sample performance in their local patient population.

## Conclusions

In conclusion, we provide a well-calibrated early prediction model for ICU-AW, identifying “high cost and high need” patients using data available within 24 h of admission. Our model can predict the probability of developing ICU-AW by day 9, serving as a communicable metric that can hopefully assist in the development of better preventive and personalized care. Further, we also make our model publicly available in an online application.

## Supplementary Information

**Additional file 1.**

## Data Availability

The datasets generated and analyzed in this study are not publicly available due to patient data confidentiality. It can be provided upon reasonable request to author Fabian Güiza Grandas.

## Footnotes

1 https://github.com/MartinWeigl/pycaleva

2 https://pypi.org/project/dcurves/

3 https://kulak.kuleuven.be/ICUAWPredictor/

4 https://shiny.posit.co/py/

5 https://shap.readthedocs.io/en/latest/

6 https://scikit-learn.org/stable/

7 https://kulak.kuleuven.be/ICUAWPredictor/

## References

[1] Afessa B, Keegan MT, Hubmayr RD, Naessens et al. Evaluating the performance of an institution using an intensive care unit benchmark. In: *Mayo Clinic Proceedings*. Elsevier. 2005; 80: pp. 174–180. 10.4065/80.2.174 15704771

[2] Brook RH (2011) Can the patient-centered outcomes research institute become rele- vant to controlling medical costs and improving value? JAMA 306(18):2020–2021 22068994 10.1001/jama.2011.1621

[3] Figueroa JF, Horneffer KE, Jha AK (2019) Disappointment in the value-based era: time for a fresh approach? JAMA 322(17):1649–1650 31596430 10.1001/jama.2019.15918

[4] Frank L, Basch E, Selby JV, Institute P-COR et al (2014) The pcori perspective on patient-centered outcomes research. JAMA 312(15):1513–1514 25167382 10.1001/jama.2014.11100

[5] Shrank WH, Rogstad TL, Parekh N (2019) Waste in the US health care system: estimated costs and potential for savings. JAMA 322(15):1501–1509 31589283 10.1001/jama.2019.13978

[6] Bruyneel A et al (2025) Frequency, financial impact, and factors associated with cost outliers in intensive care units: a cohort study in Belgium. Critical Care Science 37:e20250207 39879435 10.62675/2965-2774.20250207 PMC11805458

[7] Kelmenson DA, Held N, Allen RR et al (2017) Outcomes of intensive care unit patients with a discharge diagnosis of critical illness polyneuromyopathy: a propensity matched analysis. Crit Care Med 45(12):2055 29019851 10.1097/CCM.0000000000002763 PMC5693740

[8] Kress JP, Herridge MS. Medical and economic implications of physical dis- ability of survivorship. In: *Seminars in Respiratory and Critical Care Medicine*. Thieme Medical Publishers. 2012; 33: 339–347. 10.1055/s-0032-1321983 22875379

[9] Needham DM, Wozniak AW, Hough CL et al (2014) Risk factors for physical impairment after acute lung injury in a national, multi-center study. Am J Respir Crit Care Med 189(10):1214–1224 24716641 10.1164/rccm.201401-0158OC PMC4061900

[10] Latronico N, Herridge M, Hopkins RO et al (2017) The ICM research agenda on intensive care unit-acquired weakness. Intensive Care Med 43(9):1270–1281 28289812 10.1007/s00134-017-4757-5

[11] De Jonghe B, Bastuji-Garin S, Durand M-C et al (2007) Respiratory weakness is associated with limb weakness and delayed weaning in critical illness. Crit Care Med. 10.1097/01.ccm.0000281450.01881.d8 17855814 10.1097/01.ccm.0000281450.01881.d8

[12] Jung B, Moury PH, Mahul M et al (2016) Diaphragmatic dysfunction in patients with ICU-acquired weakness and its impact on extubation failure. Intensive Care Med 42(5):853–861 26572511 10.1007/s00134-015-4125-2

[13] Hermans G, Van Mechelen H, Clerckx B et al (2014) Acute outcomes and 1-year mortality of intensive care unit–acquired weakness. A cohort study and propensity-matched analysis. Am J Respir Crit Care Med 190(4):410–420 24825371 10.1164/rccm.201312-2257OC

[14] Vanhorebeek I, Latronico N, Berghe G (2020) Icu-acquired weakness. Intensive Care Med 46(4):637–653 32076765 10.1007/s00134-020-05944-4 PMC7224132

[15] Stevens RD, Marshall SA, Cornblath DR et al (2009) A framework for diagnosing and classify- ing intensive care unit-acquired weakness. Crit Care Med 37(10):299–308 10.1097/CCM.0b013e3181b6ef67 20046114

[16] Hermans G, Casaer MP, Clerckx B et al (2013) Effect of tolerating macronutrient deficit on the development of intensive-care unit acquired weakness: a subanalysis of the epanic trial. Lancet Respir Med 1(8):621–629 24461665 10.1016/S2213-2600(13)70183-8

[17] Casaer MP, Mesotten D, Hermans G et al (2011) Early versus late parenteral nutrition in critically ill adults. N Engl J Med 365:506–517 21714640 10.1056/NEJMoa1102662

[18] Leisman DE, Harhay MO, Lederer DJ et al (2020) Development and reporting of prediction models: guidance for authors from editors of respiratory, sleep, and critical care journals. Crit Care Med 48(5):623–633. 10.1097/CCM.0000000000004246 32141923 10.1097/CCM.0000000000004246 PMC7161722

[19] Collins GS et al (2015) Transparent reporting of a multivariable prediction model for individual prognosis or diagnosis (TRIPOD) the TRIPOD statement. Circulation 131(2):211–219 25561516 10.1161/CIRCULATIONAHA.114.014508 PMC4297220

[20] Brunello A-G, Haenggi M, Wigger O et al (2010) Usefulness of a clinical diagnosis of ICU-acquired paresis to predict outcome in patients with SIRS and acute respiratory failure. Intensive Care Med 36:66–74 19760204 10.1007/s00134-009-1645-7

[21] De Jonghe B, Sharshar T, Lefaucheur J-P et al (2002) Paresis acquired in the intensive care unit: a prospective multicenter study. JAMA 288(22):2859–2867 12472328 10.1001/jama.288.22.2859

[22] Penũelas O, Muriel A, Frutos-Vivar F et al (2018) Prediction and outcome of intensive care unit-acquired paresis. J Intensive Care Med 33(1):16–28 27080128 10.1177/0885066616643529

[23] Witteveen E, Wieske L, Sommers J et al (2020) Early predic- tion of intensive care unit–acquired weakness: a multicenter external validation study. J Intensive Care Med 35(6):595–605 29716425 10.1177/0885066618771001 PMC7222288

[24] Wulff JN, Jeppesen LE (2017) Multiple imputation by chained equations in praxis: guidelines and review. Electron J Bus Res Methods 15(1):41–56

[25] Schisterman EF et al (2008) Youden index and the optimal threshold for markers with mass at zero. Stat Med 27(2):297–315 17624866 10.1002/sim.2993 PMC2749250

[26] Nattino G, Finazzi S, Bertolini G (2014) A new calibration test and a reappraisal of the calibration belt for the assessment of prediction models based on dichotomous outcomes. Stat Med 33(14):2390–2407 24497413 10.1002/sim.6100

[27] Vickers AJ, Elkin EB (2006) Decision curve analysis: a novel method for evaluating prediction models. Med Decis Making 26(6):565–574 17099194 10.1177/0272989X06295361 PMC2577036

[28] Hill A, Fowler R, Pinto R et al (2016) Long- term outcomes and healthcare utilization following critical illness–a population- based study. Crit Care 20(1):1–10 27037030 10.1186/s13054-016-1248-y PMC4818427

[29] Dinglas VD, Friedman LA, Colantuoni E et al (2017) Muscle weakness and 5-year survival in acute respiratory distress syndrome survivors. Crit Care Med 45(3):446–453 28067712 10.1097/CCM.0000000000002208 PMC5315580

[30] Hermans G, Van Aerde N, Meersseman P et al (2019) Five-year mortality and morbidity impact of prolonged versus brief ICU stay: a propensity score matched cohort study. Thorax 74(11):1037–1045 31481633 10.1136/thoraxjnl-2018-213020

[31] Herridge MS, Moss M, Hough CL et al (2016) Recovery and outcomes after the acute respiratory distress syndrome (ARDS) in patients and their family caregivers. Intensive Care Med 42:725–738 27025938 10.1007/s00134-016-4321-8

[32] Herridge MS, Tansey CM, Matte A et al (2011) Functional disability 5 years after acute respiratory distress syndrome. N Engl J Med 364(14):1293–1304 21470008 10.1056/NEJMoa1011802

[33] Pfoh ER, Wozniak AW, Colantuoni E et al (2016) Physical declines occurring after hospital discharge in ARDS survivors: a 5-year longitudinal study. Intensive Care Med 42:1557–1566 27637716 10.1007/s00134-016-4530-1

[34] Prescott HC, Iwashyna TJ, Blackwood B et al (2019) Understanding and enhancing sepsis survivorship. Priorities for research and practice. Am J Respir Crit Care Med 200(8):972–981 31161771 10.1164/rccm.201812-2383CP PMC6794113

[35] Fan E, Dowdy DW, Colantuoni E et al (2014) Physical complications in acute lung injury survivors: a 2-year longitudinal prospective study. Crit Care Med 42(4):849 24247473 10.1097/CCM.0000000000000040 PMC3959239

[36] Hermans G, VanMechelen H, Bruyninckx F et al (2015) Predictive value for weakness and 1-year mortality of screening electrophysiology tests in the icu. Intens Care Med 41:2138–2148 10.1007/s00134-015-3979-7 26266842

[37] Herridge MS, Azoulay E (2023) Outcomes after critical illness. New Engl J Med 388(10):913–924 36884324 10.1056/NEJMra2104669

[38] Smith G (2018) Step away from stepwise. J Big Data 5(1):32. 10.1186/s40537-018-0143-6

[39] Tran A, Walsh CJ, Batt J et al (2020) A machine learning-based clinical tool for diagnosing myopathy using multi-cohort microarray expression profiles. J Transl Med 18:1–9 33256785 10.1186/s12967-020-02630-3 PMC7708151

[40] Wieske L, Witteveen E, Verhamme C et al (2014) Early prediction of intensive care unit– acquired weakness using easily available parameters: a prospective observational study. PLoS ONE 9(10):111259 10.1371/journal.pone.0111259 PMC4210178 25347675

[41] Breiman L (2001) Random forests. Mach Learn 45:5–32

[42] Parry SM, Berney S, Granger CL, Dunlop DL, Murphy L, El-Ansary D, Koopman R, Denehy L (2015) A new two-tier strength assessment approach to the diagnosis of weakness in intensive care: an observational study. Crit Care 19:52 25882719 10.1186/s13054-015-0780-5 PMC4344764

[43] Hermans G, Clerckx B, Vanhullebusch T, Segers J, Vanpee G, Robbeets C, Casaer MP, Wouters P, Gosselink R, Van den Berghe G (2012) Interobserver agreement of Medical Research Council sum-score and handgrip strength in the intensive care unit. Muscle Nerve 45:18–25 22190301 10.1002/mus.22219

[44] Kleyweg RP, van der Meché FG, Schmitz PI (1991) Interobserver agreement in the assessment of muscle strength and functional abilities in Guillain-Barré syndrome. Muscle Nerve 14(11):1103–1109 1745285 10.1002/mus.880141111

[45] Lundberg SM, Lee S-I A unified approach to interpreting model predictions. Adv Neural Informat Process Syst. 2017; 30.

