# Correction: Development and validation of a machine learning model for early prediction of intensive care unit acquired weakness

**DOI:** 10.1186/s40635-025-00817-w

**Published:** 2025-10-20

**Authors:** Felipe Kenji Nakano, Nathalie Van Aerde, Gregoire Coppens, Ilse Vanhorebeek, Celine Vens, Greet Van den Berghe, Fabian Guiza Grandas

**Affiliations:** 1 Department of Public Health and Primary Care, KU Leuven KULAK, Etienne Sabbelaan 53, 8500 Kortrijk, Belgium; 2 Itec, Imec Research Group at KU Leuven, Etienne Sabbelaan 51, 8500 Kortrijk, Belgium; 3 Department of Cellular and Molecular Medicine, Clinical Division and Laboratory of Intensive Care Medicine, KU Leuven, UZ, Herestraat 49, 3000 Louvain, Belgium

**Correction** **:** **Intensive Care Medicine Experimental (2025) 13:98** 10.1186/s40635-025-00810-3

Following publication of the original article [1], the author would like to correct a typo in the Abstract.

The sentence "Patients are diagnosed with ICU-AW if their MRC is higher than 48" should be corrected as "Patients are diagnosed with ICU-AW if their MRC is lower than 48 ".

The original article [1] has been updated.
