# Supplementary material for: Development and validation of a machine learning model for early prediction of intensive care unit acquired weakness
Source: Intensive Care Med Exp. 2025 Sep 30;13:98. doi: 10.1186/s40635-025-00810-3 (PMC12484466; doi:10.1186/s40635-025-00810-3)
Supplement: Supplementary file 1 — Additional file 1. [file 40635_2025_810_MOESM1_ESM.docx]

Development and validation of a machine learning model for early prediction of intensive care unit acquired weakness

Felipe Kenji Nakano, PhD^1,2*^***^†^***, Nathalie Van Aerde, MD, PhD^3^***^†^***, Grégoire Coppens MD, PhD^3^ , Ilse Vanhorebeek IR, PhD^3^, Celine Vens, PhD^1,2^***^†^***, Greet Van den Berghe, MD, PhD,^3^***^†^***, Fabian Güiza Grandas, IR, PhD^3^***^†^***

^1^Department of Public Health and Primary Care, KU Leuven KULAK, Etienne Sabbelaan 53, Kortrijk, 8500, Belgium.

^2^Itec, imec research group at KU Leuven, Etienne Sabbelaan 51, Kortrijk, 8500, Belgium.

^3^Department of Cellular and Molecular Medicine, Clinical Division and Laboratory of Intensive Care Medicine, KU Leuven, UZ Herestraat 49, Leuven, 3000, Belgium.

*Corresponding author(s). E-mail(s): [felipekenji.nakano@kuleuven.be;](mailto:felipekenji.nakano@kuleuven.be) Contributing authors: [vanaerde.nathalie@gmail.com;](mailto:vanaerde.nathalie@gmail.com) [gregoire.coppens@gmail.com;](mailto:gregoire.coppens@gmail.com) [ilse.vanhorebeek@kuleuven.be;](mailto:ilse.vanhorebeek@kuleuven.be) [celine.vens@kuleuven.be;](mailto:celine.vens@kuleuven.be) [greet.vandenberghe@kuleuven.be;](mailto:greet.vandenberghe@kuleuven.be) [fabian.guiza@kuleuven.be;](mailto:fabian.guiza@kuleuven.be)

***^†^***These authors contributed equally to this work.

[1 Supplementary Material 2](#_Toc1075492903)

[1.1 Most relevant descriptors 2](#_Toc1354263138)

[1.2 Online tool 2](#_Toc1088599436)

[1.3 Calibration belt logistic regression 5](#_Toc102131480)

[1.4 Calibration belt random forest using only APACHE II 5](#_Toc373073706)

[1.5 Calibration belt random forest using only SOFA PaO2/FiO2 score 6](#_Toc62016396)

[1.6 Evaluation metrics considering random forest, logistic regression, random forest using only APACHE II and random forest using only SOFA PaO2/FiO2 score 7](#_Toc1981248672)

[1.7 Histograms of laboratory features 7](#_Toc1193688089)

[1.8 Partial dependency plot 12](#_Toc2106316225)

[2. Randomized Clinical Trial inclusion and exclusion criteria 13](#_Toc1028043390)

[2.1 Inclusion Criteria 13](#_Toc1370735251)

[2.1 Exclusion Criteria 13](#_Toc208811315)

[3. Key reporting metrics for prediction models 14](#_Toc1338153350)

[References 15](#_Toc1240868006)

# 1 Supplementary Material

## 1.1 Most relevant descriptors

The list below contains all descriptors ranked according to their relevance, as determined by the random forest:

1. APACHEII
2. Creatinine
3. SOFA PaO2/FiO2
4. C-Reactive protein
5. Bilirubin
6. BMI
7. Age
8. Morning glycemia
9. Admission glycemia
10. Sepsis
11. Infection upon admission
12. Steroids
13. Gender
14. Malignancy
15. Diabetes
16. Mechanical ventilation
17. Dialysis

## 1.2 Online tool

Our online tool may be used in two modes: i) single patient and ii) multiple patients. In the first mode, users manually input the information about a patient and the tool reports the chance of developing ICU-AW by day 9 (prediction) and the interpretation of the prediction using SHAP^[[1]](#footnote-3248)^([1](bookmark://_bookmark1)). Further, a global explanation, where the model used by the online tool is explained as a whole, is also available. This mode is portrayed in Supplementary Material Figure 1.

The second mode allows the user to upload data related to multiple patients. The tool outputs the prediction regarding each patient and also the AUROC, if the diagnosis of ICU-AW is given as input. Supplementary Material Figure [2](bookmark://_bookmark6) depicts this functionality.

To develop our online application, we have used the same resources listed in Section 2 and also the libraries Shiny 0.6^[[2]](#footnote-21115)^, Shap 0.43^[[3]](#footnote-19068)^ and Scikit-learn 1.3.2^[[4]](#footnote-23266)^.


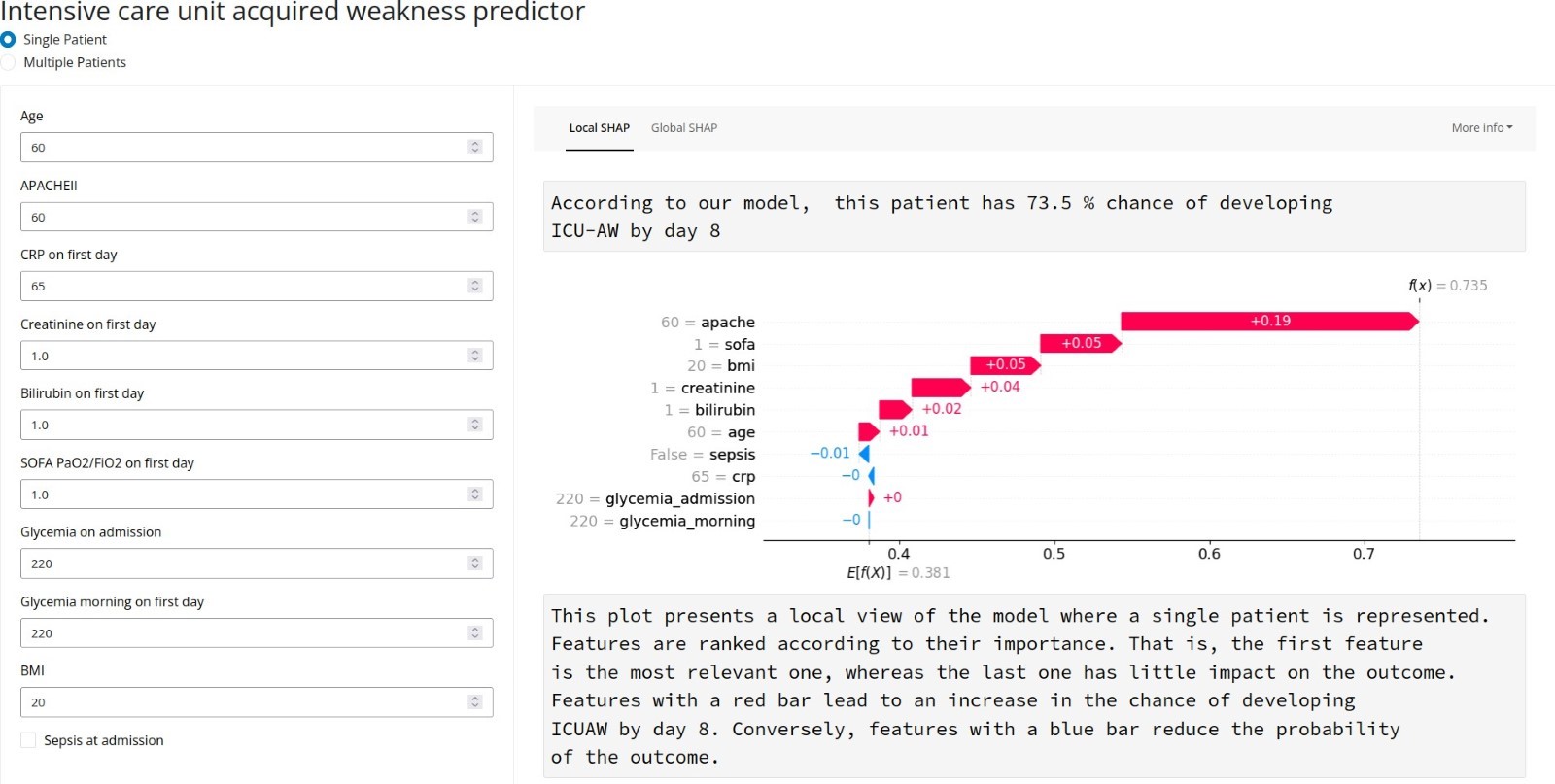


**Figure 1.** Online application reporting the prediction on a single patient and its interpretation.


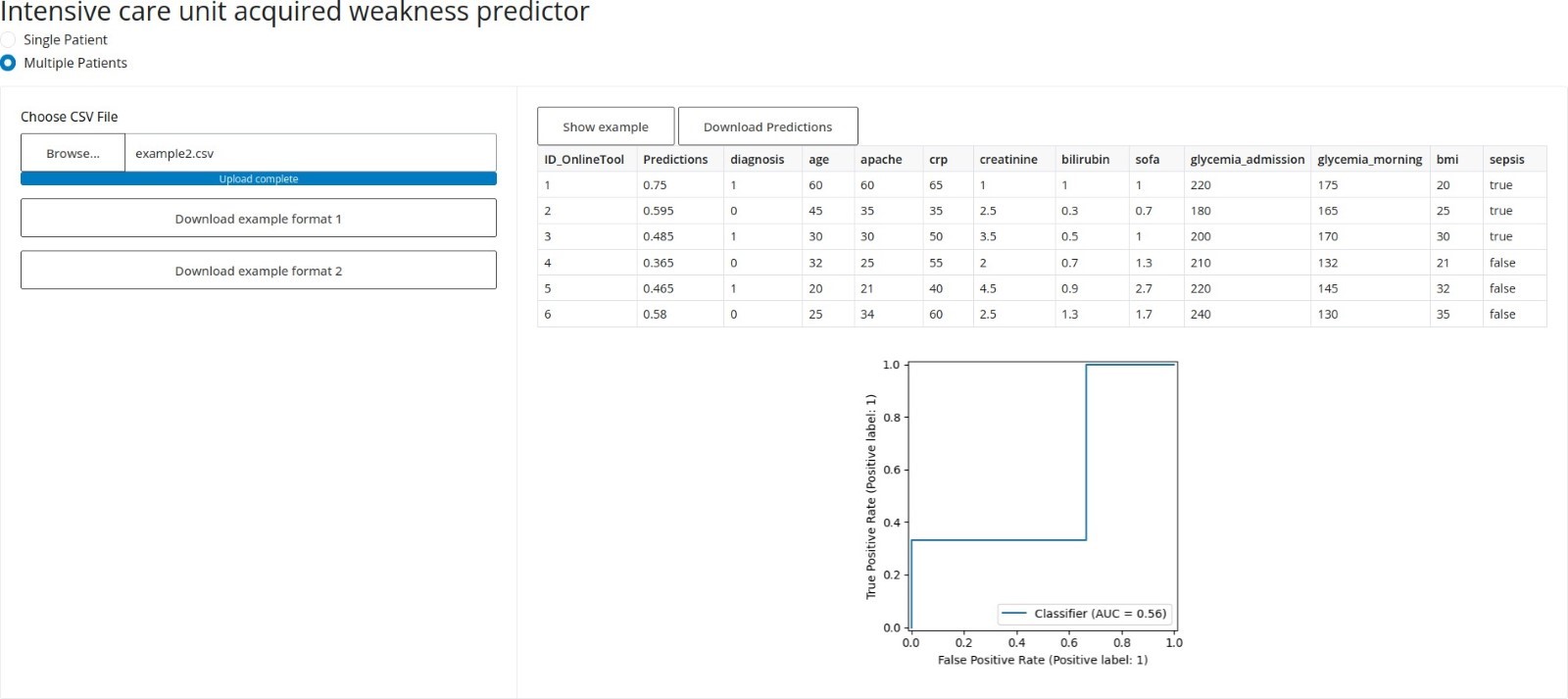


**Figure 2.** Online application reporting the prediction of multiple patients and its performance according to AUROC.


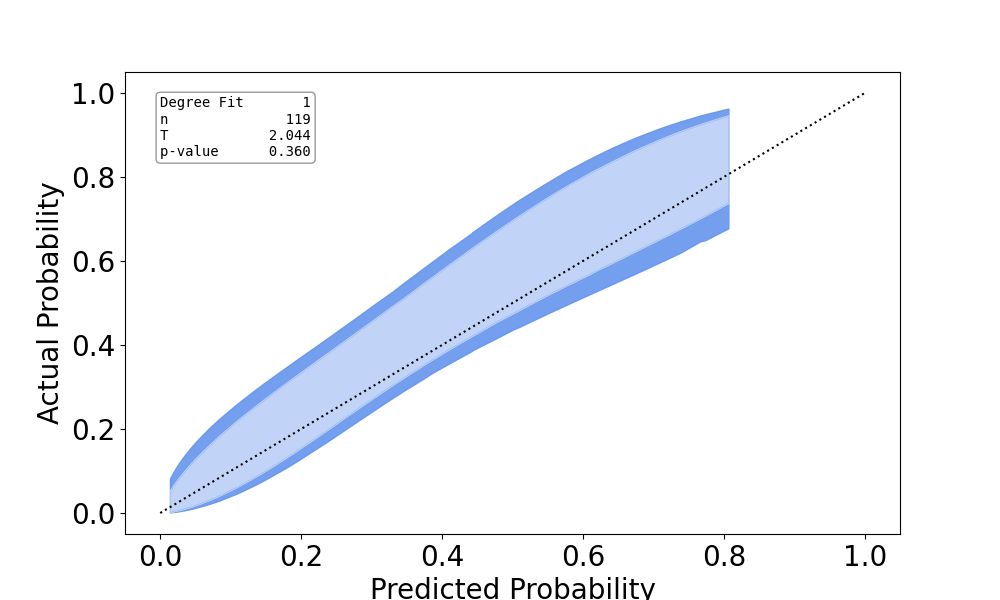


**Figure 3.** Calibration belt considering 5-fold cross-validation for the random forest available in the online application. The x-axis contains the probabilities predicted by the random forest, whereas the y-axis contains the expected output. Once again, no evidence of miscalibration can be found since the diagonal is within the blue region.


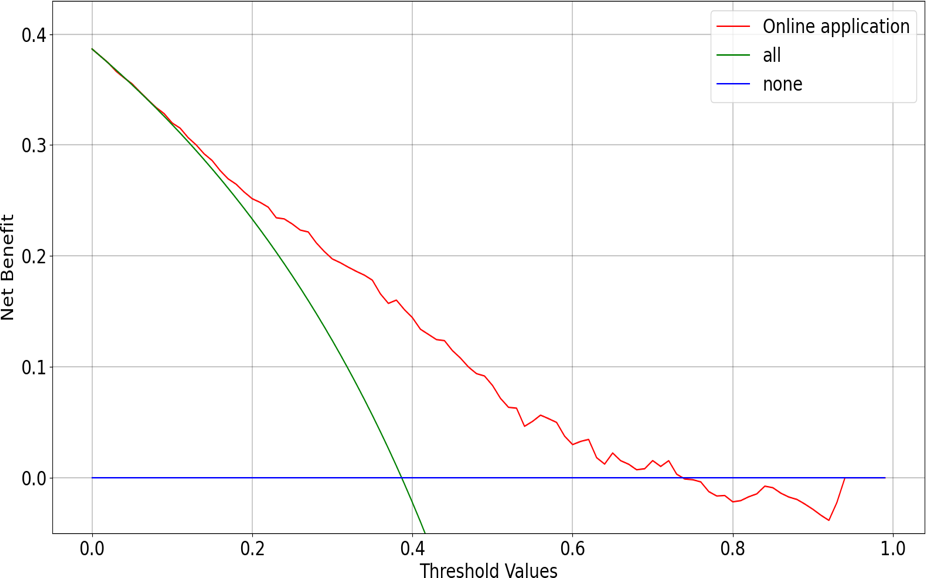


**Figure 4.** Decision curve using the random forest available in the online application considering 5-fold cross-validation. The x-axis contains the probabilities predicted by the random forest, whereas the y- axis contains the clinical usefulness. Identically to the random forest using all descriptors, clinical usefulness is observed in thresholds ranging from 10% to 70%.

## 1.3 Calibration belt logistic regression


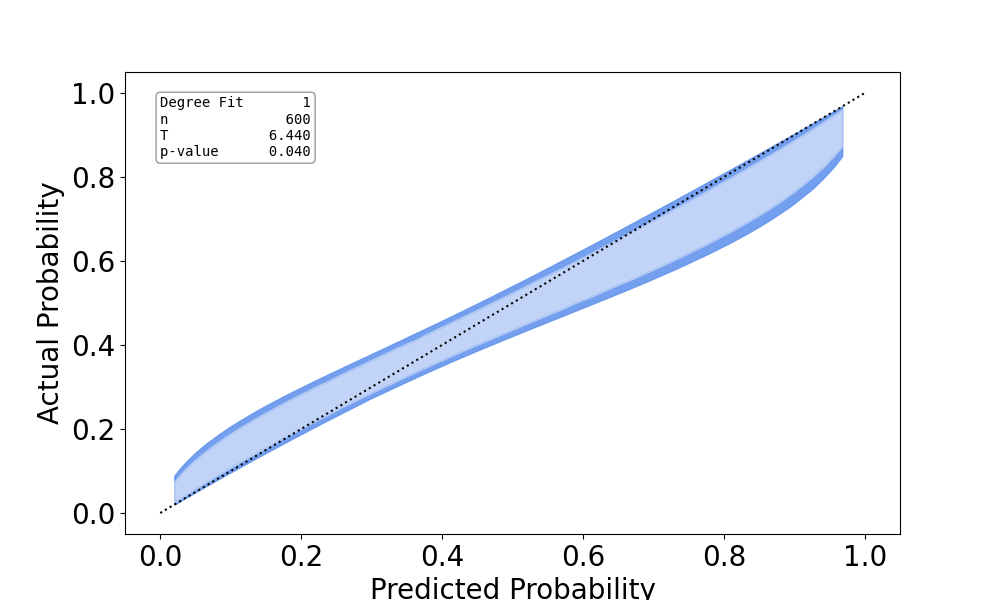


**Figure 5.** Calibration belt considering 5-fold cross-validation and the logistic regression. The x-axis contains the probabilities predicted by the logistic regression, whereas the y-axis contains the expected output. In this case, no evidence of miscalibration can be found if the diagonal is within the blue region.

## 1.4 Calibration belt random forest using only APACHE II


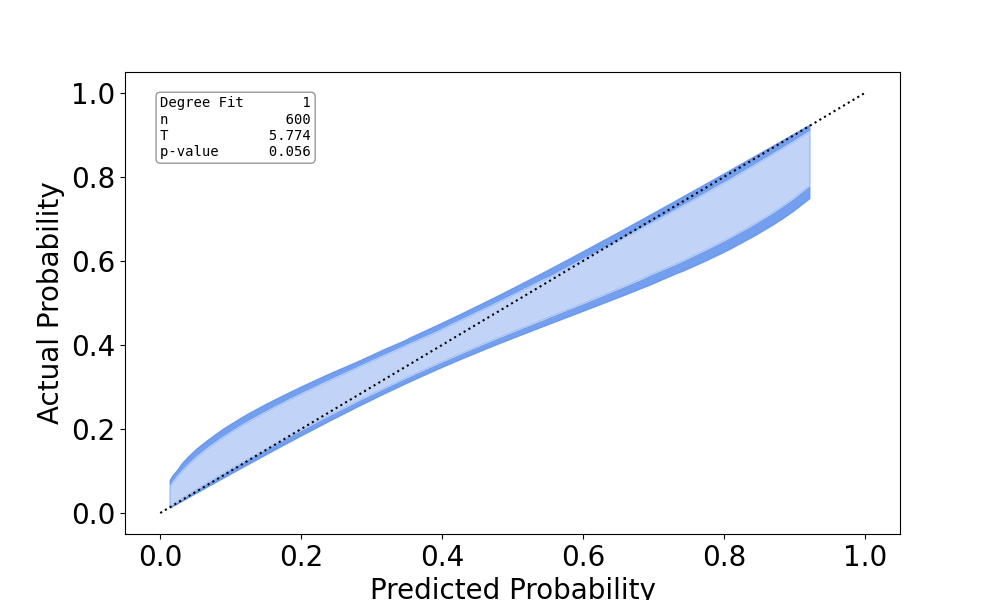


**Figure 6.** Calibration belt considering 5-fold cross-validation and the random forest using only APACHE as a descriptor. The x-axis contains the probabilities predicted by the random forest whereas the y-axis contains the expected output. In this

case, the evidence of miscalibration cannot be rejected, assuming a p-value of 0.05.

## 1.5 Calibration belt random forest using only SOFA PaO2/FiO2 score


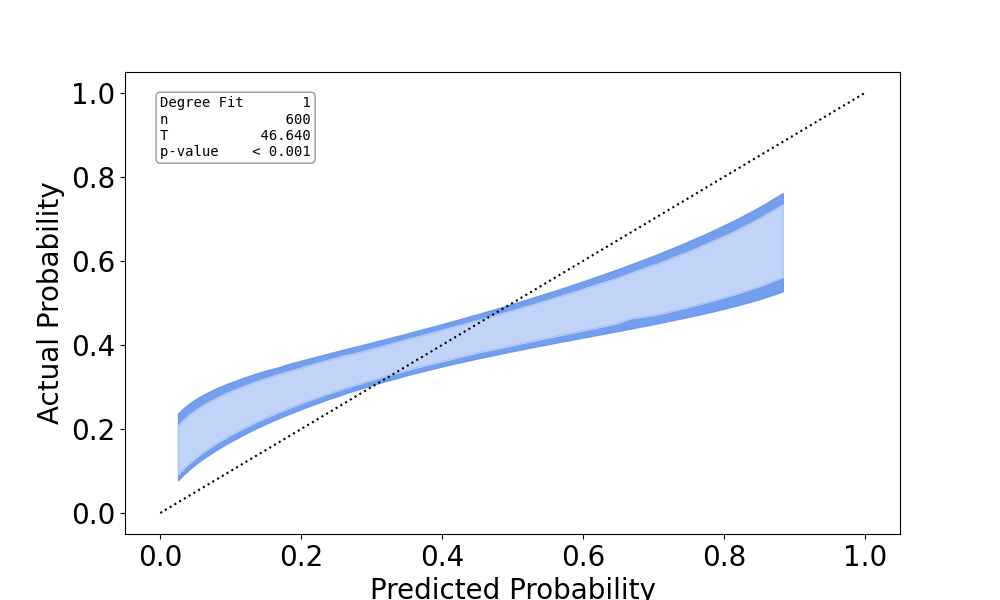
**Figure 7.** Calibration belt considering 5-fold cross-validation and the random forest using only SOFA PaO2/FiO2 score as a descriptor. The x-axis contains the probabilities predicted by the random forest, whereas the y-axis contains the expected output. In this case, the evidence of miscalibration cannot be rejected, assuming a p-value of 0.05.

## 1.6 Evaluation metrics considering random forest, logistic regression, random forest using only APACHE II and random forest using only SOFA PaO2/FiO2 score

| Metric | RF | LR | RF APACHE II | RF SOFA PaO2/FiO2 score |
| --- | --- | --- | --- | --- |
| AUROC | 76 | 74 | 70 | 62 |
| Specificity | 62 | 77 | 63 | 66 |
| Sensitivity | 79 | 66 | 67 | 54 |

**Table 1**. Average results obtained on 100 times 5-fold cross validation. RF stands for the random forest with all descriptors, LR for logistic regression, RF APACHE II for the random forest using only APACHE II as a single descriptor and RF SOFA score its counterpart using only the SOFA PaO2/FiO2 score.

## 1.7 Histograms of laboratory features


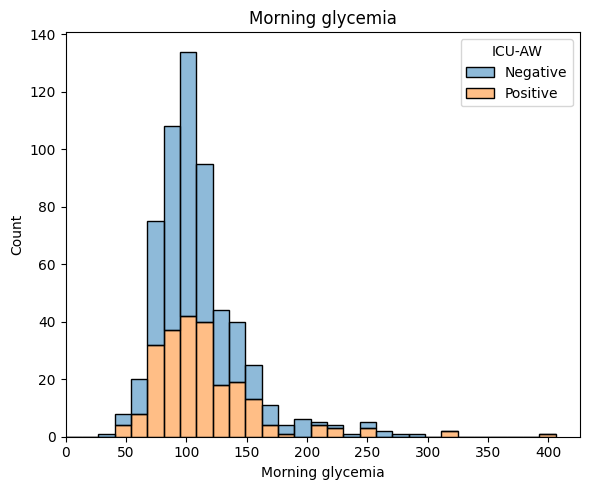


**Figure 8.** Histogram containing morning glycemia (x-axis) versus density (y-axis), with values stratified by ICU-AW diagnosis.
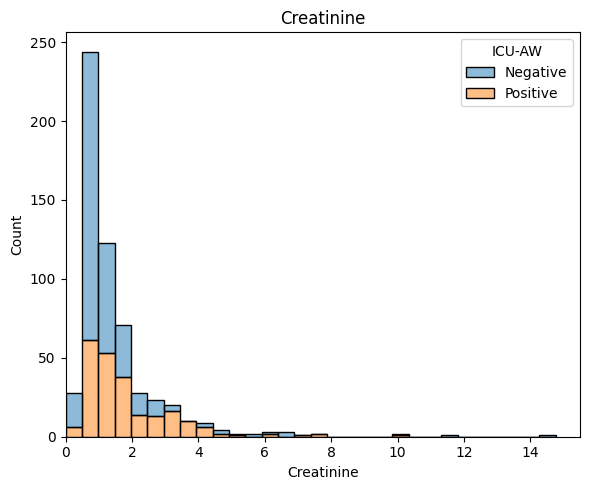


**Figure 9.** Histogram containing creatinine (x-axis) versus density (y-axis), with values stratified by ICU-AW diagnosis.


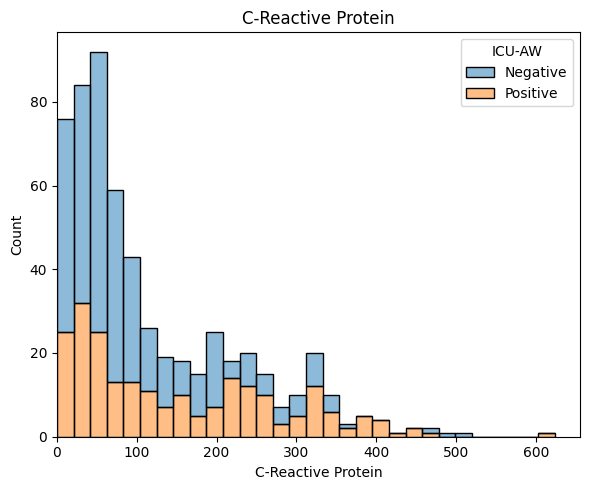


**Figure 10.** Histogram containing C-Reactive Protein (x-axis) versus density (y-axis), with values stratified by ICU-AW diagnosis.


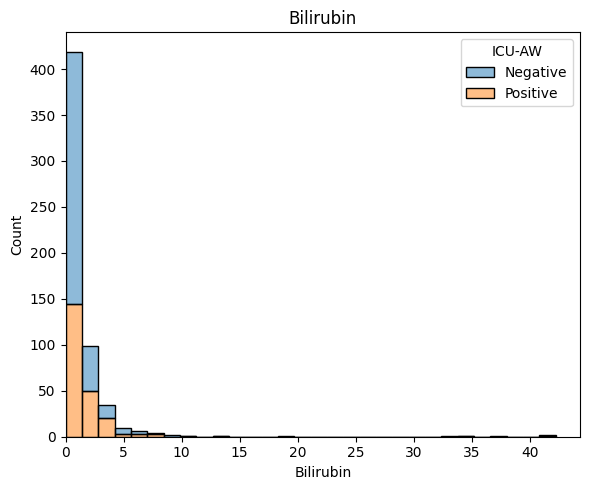


**Figure 11.** Histogram containing Bilirubin (x-axis) versus density (y-axis), with values stratified by ICU-AW diagnosis.


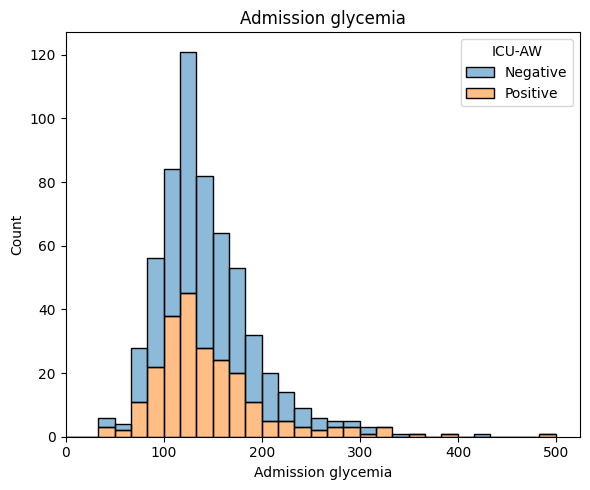


**Figure 12.** Histogram containing admission glycemia (x-axis) versus density (y-axis), with values stratified by ICU-acquired weakness (ICU-AW) diagnosis.

## 1.8 Partial dependency plot


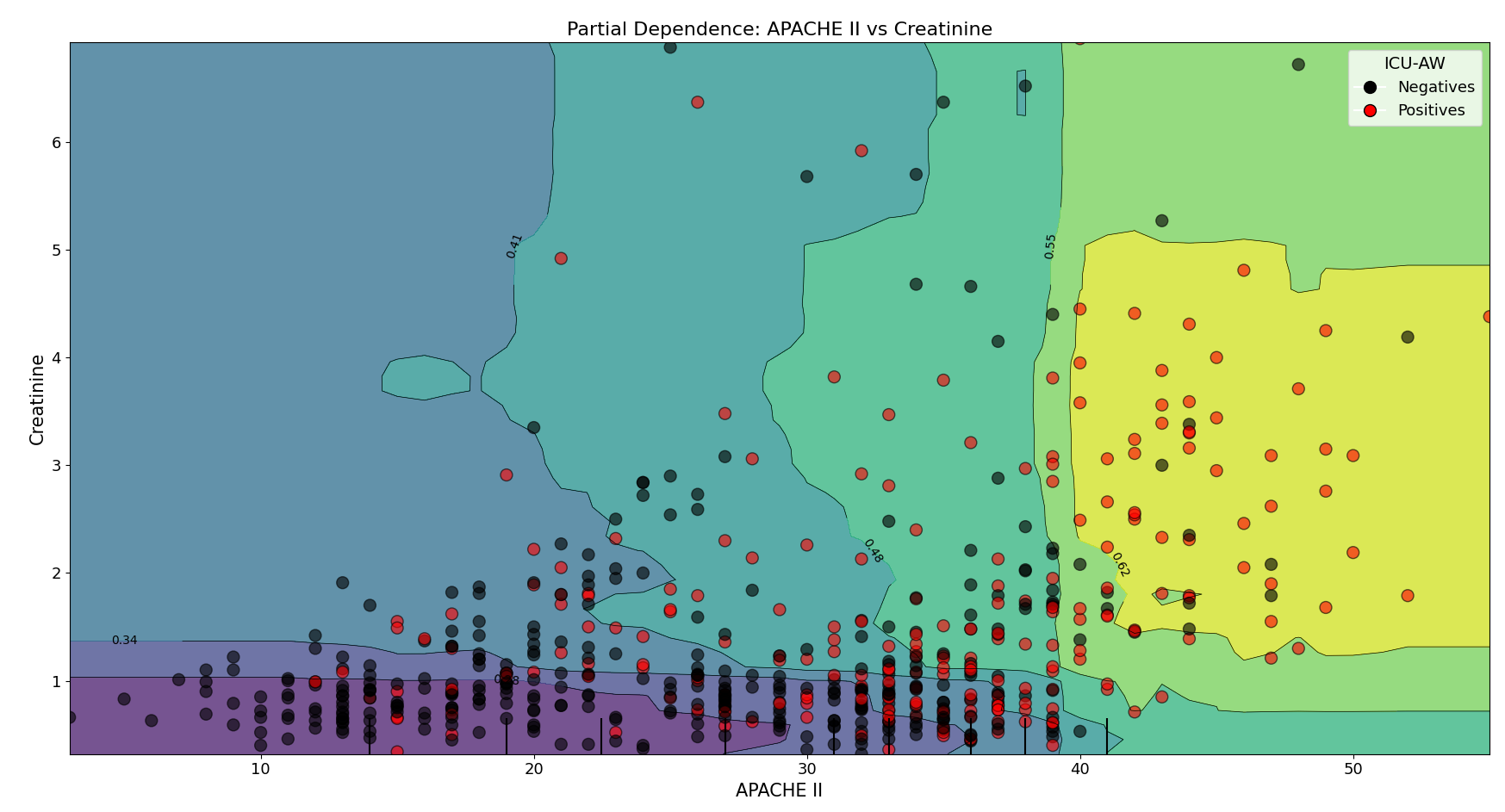
**Figure 13.** Partial dependency plot containing admission APACHE II (x-axis) versus creatinine (y-axis) generated using the random forest. Purple tones indicate lower predictive probability of ICU-AW, whereas yellowish tones indicate higher predictive probabilities. Red and black dots correspond to patients diagnosed and non-diagnosed with ICU-AW, respectively.

# 2. Randomized Clinical Trial inclusion and exclusion criteria

## 2.1 Inclusion Criteria

1. Patients admitted to any of the five intensive care units
2. Older than 18 years
3. Nutritional risk screening score (NRS) higher or equal to three upon ICU admission

## 2.1 Exclusion Criteria

1. Patients with a do not resuscitate (DNR) code or moribund at the time of ICU admission
2. Patients already enrolled in another trial
3. Patients transferred from another intensive care unit with an established nutritional therapy
4. Patients suffering from ketoacidotic or hyperosmolar coma on admission
5. Patients with a body mass index (BMI) below 17 kg/m^2
6. Short bowel syndrome
7. Patients known to be pregnant or nursing
8. Patients on mechanical ventilation at home
9. NRS score lower than three
10. Patient readmitted to ICU after randomization to the EPaNIC trial.
11. Patient not critically ill on admission. (No clinical indication for central intravenous catheter or patient ready for oral nutrition on admission)

# 3. Key reporting metrics for prediction models

In Table 2, we present the “Key Reporting Metrics” as recommended in (2).

| Domain | Key Reporting Elements |
| --- | --- |
| Data source | Section 2.1 - EPaNIC-trial (NCT00512122, N = 4640). |
| Participants | Section 2.1 - EPaNIC-trial (NCT00512122, N = 4640). |
| Outcome | Section 2.2 - ICU-AW measured using the medical research council sum scale. |
| Predictors | Section 2.3 - baseline factors (age, diabetes, BMI as a continuous value, malignancy, sepsis, gender and preadmission dialysis). Admission factors were also included, such as randomization strategy in the original trial, infection upon admission, APACHE II, admission glycemia and factors reflecting the first 24 hours of ICU-stay (laboratory values as markers for severity of individual organ failure i.e., total serum bilirubin, CRP, serum creatinine, glycemia levels at admission and in the morning after, and SOFA PaO2/FiO2) and treatment factors (mechanical ventilation and corticosteroid administration on the first day). All features were used as input in their original form. |
| Missing data | Report in Table 1. Missing values were imputed using multiple chained equations with 31 iterations. |
| Model specification | Section 2.3 - We used a random forest, with 150 trees, and a logistic regression with l2 normalization. |
| Model structure | Not applicable |
| Validation | Section 2.4 - Internally validated. We reported the AUROC averaged over 100 times 5-fold cross validation. We also present decision curves and calibration belts. |
| Model performance | Section 3.2 - The best model was the random forest which achieved 76% AUROC. |

Table 2. Key reporting metrics.

# **References**

1. Lundberg, S.M., Lee, S.-I.: A unified approach to interpreting model predictions. Advances in neural information processing systems 30 (2017)
2. Leisman DE, Harhay MO, Lederer DJ, et al: Development and reporting of prediction models: guidance for authors from editors of respiratory, sleep, and critical care journals. *Crit Care Med* 2020; 48(5):623-633. doi: 10.1097/CCM.0000000000004246. PMID: 32141923; PMCID: PMC7161722.

1. https://shap.readthedocs.io/en/latest/ [↑](#footnote-ref-3248)
2. https://shiny.posit.co/py/ [↑](#footnote-ref-21115)
3. https://shap.readthedocs.io/en/latest/ [↑](#footnote-ref-19068)
4. https://scikit-learn.org/stable/ [↑](#footnote-ref-23266)
